# Rethinking physical activity communication: using focus groups to understand women’s goals, values, and beliefs to improve public health

**DOI:** 10.1186/s12889-017-4361-1

**Published:** 2017-05-18

**Authors:** Michelle Segar, Jennifer M. Taber, Heather Patrick, Chan L. Thai, April Oh

**Affiliations:** 1 Sport, Health, and Activity Research and Policy (SHARP) Center, University of Michigan, Ann Arbor, MI 48109 USA; 2 Behavioral Research Program, National Cancer Institute, National Institutes of Health, 9609 Medical Center Drive, Rockville, MD 20850 USA; 3 Behavioral Science, Carrot Sense, Inc., 1600 Seaport Blvd, Suite 150, Redwood City, CA 94063 USA; 4 Department of Communication, Santa Clara University, 500 El Camino Real, Santa Clara, CA 95053 USA; 5 Health Communication and Informatics Research Branch, Behavioral Research Program, National Cancer Institute, 9609 Medical Center Drive, Rockville, 20850 MD USA

**Keywords:** Physical activity, Exercise, Self-determination theory, Goals, Values, Priorities, Happiness, Communication, Messaging, Women

## Abstract

**Background:**

Communication about physical activity (PA) frames PA and influences what it means to people, including the role it plays in their lives. To the extent that PA messages can be designed to reflect outcomes that are relevant to what people most value experiencing and achieving in their daily lives, the more compelling and effective they will be. Aligned with self-determination theory, this study investigated *proximal* goals and values that are salient in everyday life and how they could be leveraged through new messaging to better support PA participation among women. The present study was designed to examine the nature of women’s daily goals and priorities and investigate women’s PA beliefs, feelings, and experiences, in order to identify how PA may compete with or facilitate women’s daily goals and priorities. Preliminary recommendations are proposed for designing new PA messages that align PA with women’s daily goals and desired experiences to better motivate participation.

**Methods:**

Eight focus groups were conducted with White, Black, and Hispanic/Latina women aged 22–49, stratified by amount of self-reported PA (29 low active participants, 11 high active participants). Respondents discussed their goals, values, and daily priorities along with beliefs, feelings about and experiences being physically active. Data were collected, coded, and analyzed using a thematic analysis strategy to identify emergent themes.

**Results:**

Many of the goals and values that both low and high active participants discussed as desiring and valuing map on to key principles of self-determination theory. However, the discussions among low active participants suggested that their beliefs, feelings, experiences, and definitions of PA were in conflict with their proximal goals, values, and priorities, also undermining their psychological needs for autonomy, competence, and relatedness.

**Conclusions:**

Findings from this study can be used to inform and evaluate new physical activity communication strategies that leverage more proximal goals, values, and experiences of happiness and success to better motivate PA among ethnically diverse low active women. Specifically, this research suggests a need to address how women’s daily goals and desired experiences may undermine PA participation, in addition to framing PA as facilitating rather than competing with their daily priorities and desired leisure-time experiences.

## Background

Engaging in regular physical activity (PA) is critical for fostering health and well-being and preventing a variety of conditions and illnesses including overweight and obesity, diabetes, heart disease, and several cancers [1, 2]. Women are less physically active than men and as they age their PA participation decreases [1, 3]. Furthermore, in the U.S., compared to whites, African American and Latina women are disproportionately inactive [1] and at greater risk of many chronic illnesses [4]. Thus, a public health priority is to increase and maintain levels of PA over the life course among ethnically diverse women. PA public health messaging and social marketing frame physical activity and influence what it means to people, including their PA beliefs, perceptions, and definitions and the role it plays in their lives [5].

To date most messages promoting PA—from public service announcements to patient-provider communication to apps—have been developed based on the assumptions that (a) people value their health and (b) this value is *compelling enough* to motivate daily decisions and behavior. These messages have framed health behaviors like PA primarily as a means to achieve health-related goals such as weight loss, disease prevention, and healthy aging [6]. In fact, individuals frequently report being active specifically to achieve better health and disease prevention goals [7]. However, recent research suggests that the health benefits of PA that are commonly featured in public health communication and promotion actually predict less participation than more immediately experienced emotional benefits from PA such as improved mood and enjoyment [8–10]. Furthermore, despite decades of research on barriers and facilitators of physical activity, most interventions do not promote increased physical activity that is sustained. Thus, there is a need to investigate how to make physical activity more relevant and meaningful so individuals will be more compelled to sustain it. To the extent that public health messages can be designed to better reflect people’s daily priorities and desired experiences the more relevant and effective such promotions will be [5, 11].

Several behavioral theories address goals and values but researchers have rarely leveraged these principles to study and promote health behaviors such as PA. Self-Determination Theory (SDT), however, is one such theory that uses these principles to investigate health behaviors. SDT is a broad theory of human motivation that addresses the intersection of (1) motivational quality (i.e., the extent to which individuals engage in behaviors primarily because of external or internal pressures, a sense of congruency between the behavior and their identity, and/or the behavior is inherently valuable in its own right), (2) the role of the social environment in supporting or thwarting the basic psychological needs that cultivate higher-quality, internalized motivation, and (3) the impact of individuals’ goals, priorities, and values in supporting sustained behavioral engagement [12, 13].

To date, much of the research applying SDT to health and health behavior change has focused on motivational quality and the social environment in promoting behavioral initiation and maintenance. This body of research has demonstrated that when individuals have higher-quality, internalized motivation for behaviors like PA, they are more likely to engage in those behaviors, persisting through failures, and maintaining behavior change over time [14–16].

SDT-based interventions have been designed to create social environments within the intervention context that facilitate the internalization of motivation by supporting psychological needs for autonomy (i.e., the need to self-direct behavior change), competence (i.e., the need to feel capable of achieving desired outcomes), and relatedness (i.e., the need to feel that others understand and are empathetic toward the challenges inherent in behavior change). Across a range of behaviors including tobacco cessation, physical activity, weight management, medication adherence, dietary change, and oral health, SDT-based interventions have demonstrated efficacy for supporting psychological needs, facilitating internalization of motivation, and eliciting both short-term and sustained behavior change [17–19].

In general, SDT has identified *broad* goals and values relevant to health behavior change (e.g., health, social connection, image and appearance, fame), and to date a handful of SDT studies have examined the role of these broader goals in health behavior [8, 20, 21]. Yet, SDT research has not examined more *proximal* goals and priorities (e.g., items on the daily “to do” list) that are salient in everyday life nor how best to leverage these daily goals and priorities in PA communication strategies to support behavior change. Thus, there is a need to conduct formative, qualitative research to explore these issues.

The present qualitative study employed focus groups designed to (1) examine the nature of women’s goals and daily priorities and (2) examine women’s beliefs and feelings about and experiences with PA, and how they may compete with or facilitate their daily goals and priorities. Based on the findings, we propose preliminary recommendations about how women’s more proximal goals, priorities, and desired experiences could be integrated into public health messaging to better foster the internalization of the value of PA and motivate physical activity among ethnically diverse women.

## Methods

### Sample and recruitment

Participants were recruited from August to October of 2014 in the greater Washington DC area using fliers, Craigslist (i.e., classified advertisements website), newspaper advertisements, recruiting databases, and web and social media outlets such as GoogleAds, Facebook advertisements, and blog sites. Interested participants were screened over the telephone to determine eligibility based on gender (female only), age (between 20 to 50), race/ethnicity, and days and minutes of self-reported PA per week. Minutes of PA was assessed with the questions, “Physical activity is activity where you move and increase your heart rate above its resting rate, and includes things such as using gym equipment, walking, swimming, or biking. During a typical week, how many days are you physically active?” and “When you are physically active, about how many minutes do you spend being active?” Participants were categorized as ‘low active’ if they reported being physically active less than three times a week *or* for less than 120 min in a week and ‘high active’ if they reported more than that. The sampling strategy over-sampled participants in the low active category as those are the individuals for whom public health messaging could be better targeted and for whom other goals and values may be more salient than health and/or PA. Because our primary goal was to understand the beliefs and goals of women who were not regularly physically active, our criterion for this cut off was based on past physical activity guidelines (e.g., 3 days per week) to ensure that these participants were actually ‘low actives” and not on the cusp of being ‘high actives.’

We recruited based on race/ethnicity to ensure a more representative sample, and we further stratified the low active groups by race/ethnicity because focus groups tend to yield more candid responses when they are matched on demographic and other characteristics. Given the small number of high-active women recruited, these women participated in one of two groups that were not stratified by race/ethnicity. Each focus group had 4–7 participants, with 40 women participating in total (29 low active and 11 high active). Characteristics of participants are presented in Table 1, stratified by PA level as reported during the screening. Participants were on average 36 years old with an average BMI of 30.1. Not surprisingly, high active participants had lower mean BMI’s (26.1) than low active participants (31.7). Over 95% of participants had at least some college education, with 37.5% White, 32.5% Hispanic/Latina, and 30.0% Black. Participants were compensated $30 for focus group participation. Each focus group lasted approximately 90 min.

**Table 1:** Demographic factors by physical activity levels

	High active (*n* = 11)	Low active (*n* = 29)	Total (*N* = 40)
Demographic characteristics	*M* (*SD*)	*M* (*SD*)	*M* (*SD*)
Age in years	37.5 (6.7; range = 26–45)	36.0 (7.8; range = 22–49)	36.4 (7.5)
Body mass index	26.1 (6.3; range = 16.3–39.5)^a^	31.7 (10.1; range = 18.5–58.5)^b^	30.1 (9.4; range = 16.3–58.5)^ab^
	*n* (%)	*n* (%)	*n* (%)
Race/ethnicity			
White	5 (45.5)	10 (34.5)	15 (37.5)
Hispanic/Latina	5 (45.5)	8 (27.6)	13 (32.5)
Black	1 (9.1)	11 (37.9)	12 (30.0)
Education			
High school or less	2 (18.2)	3 (10.3)	5 (12.5)
Some college or associate’s degree	4 (36.4)	14 (48.3)	18 (45.0)
College graduate	5 (45.5)	11 (37.9)	16 (40.0)
Master’s degree	0 (0)	1 (3.4)	1 (2.5)
PA	*M* (*SD*)	*M* (*SD*)	*M* (*SD*)
Minutes of PA per week as reported during telephone screening	238.2 (138.0; range = 90–480)	30.6 (41.1; range = 0–120)^c^	89.0 (123.3)^c^
Minutes of PA per week as reported on survey	262.5 (245.4; range = 0–840)^a^	77.3 (145.5; range = 0–600)^b^	132.9 (197.6)^ab^
	*n* (%)	*n* (%)	*n* (%)
Met recommended 150 min/week of PA based on screener	7 (63.6)	0 (0)^c^	7 (18.0)^c^
Met recommended 150 min/week of PA based on survey	7 (58.3)^a^	2 (3.6)^b^	9 (22.5)^ab^

^a^These numbers include data from one participant who completed the survey but left within the first 15 min of the focus group due to illness. This participant is not included in the overall analysis or in data from the telephone screening

^b^Data are missing from 1 participant who arrived late and did not complete the survey

^c^Data are missing from 1 participant who completed the screener twice and thus had inconsistent data on this question

### Focus groups process

Eight focus groups were held in October 2014. Seven were held at a focus group test facility in Rockville, Maryland, and one was held at a church in a Latino community (Langley Park, MD) in an effort to recruit Latina women. All focus groups were led by one of three trained moderators selected to match the ethnicity and activity levels of each focus group participants. At the start of each focus group, participants provided informed consent. Next, participants completed a short questionnaire to record demographic information (i.e., height, weight, race/ethnicity, and education) and PA levels. PA was assessed with the items modified from HINTS measures (i.e., Health Information National Trends Survey) [22], “In a typical week, how many days do you do any PA or exercise of at least moderate intensity, such as brisk walking, bicycling at a regular pace, and swimming at a regular pace?” and “On the days that you do any PA or exercise of at least moderate intensity, how long do you typically do these activities?” Moderators followed a focus group guide and engaged participants in approximately 90-min discussions. Each focus group was audio-taped (with participant permission) and transcribed. The study was approved by The Office for Human Research Protections #12257.

### Focus group guide

The focus group guide aimed to elicit (1) Experiences from and aspects of daily life that drive happiness and success; (2) PA beliefs, feelings, and experiences; and (3) the ways in which PA beliefs, feelings, and experiences interact with, support, and/or undermine the drivers of happiness and success. While there are several different ways to characterize people’s goals and values, for this study our questions focused on the immediate goals and priorities related to women’s daily lives, and operationalized them as the things that contribute to daily feelings of happiness and success. This is consistent with the extant literature that has characterized these experiences and outcomes as common core values and goals toward which most people aspire and are often used by industry to market products and services [23–28].

The focus groups started broadly with questions about what contributes to participants’ happiness (e.g., What in your life makes you happy? If you could experience your perfect day, what would it look like?) and success (e.g., What in your life makes you feel successful?). Following this first set of questions, the moderator inquired about participants’ PA beliefs, feelings, definitions, and barriers (e.g., When you think about exercising or being physically active, what is the first thing that pops into your mind? What counts as PA?). Participants were also asked about their prioritization of PA (If you were going to prioritize exercise/PA in your life, where would it sit on your list of priorities? Why?).

### Analyses

Coding for themes that emerged in the data [29], two investigators (MS and JT) independently examined the data to identify commonalities and meaning within each of the following question areas: Experiences from and aspects of daily life promoting 1) happiness, 2) success, and 3) PA beliefs, feelings, definitions, and barriers. The two coders discussed their perspectives on the meaning of participants’ comments within each question area (e.g., ‘Beliefs and Feelings about PA”). Through consensus, they categorized similar comments into micro-level themes (e.g., negative thoughts about exercise *before going*, memories about negative experiences from *past* exercise). The micro-themes were then evaluated for potential high-level interconnections and meanings in order to aggregate them into the final higher-order themes (e.g., negative affective recall and forecasting) that are discussed in this paper. This process was iterative and involved moving back and forth between the raw data and micro-themes multiple times to ensure that the participants’ experiences were accurately captured. Any differences between participants in the low and high active focus groups were also noted during coding and analysis, and are relayed in the paper.

## Results

The results are organized by the question categories used to collect and analyze data and describe the higher-order themes within each. The first section reports findings related to what participants reported made them feel happy. The second section reflects participants’ responses to what makes them feel successful, and the third section contains findings related to participants' PA beliefs, feelings, definitions, and experiences. Although no differences emerged between low active and high active groups regarding happiness and success there were some differences regarding PA beliefs, feelings, and experiences that are noted.

### Happiness

Four themes emerged characterizing women’s daily sources of happiness: (1) Connecting with others, (2) Being of service to others, (3) Participating in leisure activities and hobbies, and 4) Feeling relaxed and free from daily pressures.

#### Connecting with others

The connecting with others theme related to relationships and spending time with family, friends, and even pets. Discussions referred to being with people physically, (e.g., “*doing activities with family*”), the importance of the relationship itself (e.g., “*I am a newlywed. Just being married is a total[ly] different experience for me, it's making me happy*”), and the need to feel close to and connected with others (e.g., “*Connecting is very important. If I don't see my roommates or my boyfriend, or talk to my mom all day, I feel like something is missing*”). Only one participant mentioned that connecting online with others through social media was a source of happiness.

#### Being of service

Participants reported deriving daily happiness through the experience of helping and serving others in different aspects of their lives. Examples included providing for their families financially or contributing to their children’s success (e.g., “*What makes me happy on a daily basis is providing for my family. I love seeing the smile on their faces [when] I cook*”), as well as volunteer work, and paid work.

#### Participating in leisure activities and hobbies

Participants mentioned feeling happy when participating in a variety of leisure activities and hobbies (e.g., shopping, cooking, reading). Some talked about these activities as a conduit for relaxation while others talked about them as energizing (e.g., listening to music). In addition, being physically active was frequently mentioned as an enjoyable leisure activity, including walking, playing volleyball, or working out among high and low active participants.

#### Feeling relaxed and free from pressures

When they felt relaxed and free from pressures participants said they felt happy**.** They emphasized their need to relax and recover from their daily demands and busy lives:

“*Getting to just relax. Not worry about time, schedules or anything, because we are constantly on a schedule.”*

Relaxation included specific activities such as taking naps, meditating, and simply having time off. For example, one participant said “*unwinding at the end of the day*” makes her happy while another one said “*long weekends make me happy, with no work*.” Others noted alone time as a way to relax: “*Probably after all my kids have gone to school, and I come home and I just have silence for a couple of hours..*.”

Getting relief from pressures that are associated with daily roles and responsibilities was a key aspect of relaxing. Participants mentioned a perfect day would consist of waking up late, not worrying about a schedule, and not having to do their typical daily responsibilities, such as laundry and cooking.

In sum, happiness appears to be generally derived from experiences related to spending time in activities that women autonomously choose to do, especially those that deliver relaxation and a respite from daily demands, as well as connecting with others and being of service.

### Success

Three themes arose concerning contributors to success: (1) Contributing to the success and happiness of others, (2) Accomplishing goals, and (3) Professional achievement. Across these themes, feelings of success generally came from helping others accomplish their goals, accomplishing their own goals, and from seeing the impact of their efforts on others.

#### Contributing to the success and happiness of others

Respondents discussed contributing to the improvement of others’ lives, including serving different people, often their children:

*“..that my kids are doing well. One's already graduated from the university, and he is engaged to be married. That they are on a good path makes me feel successful.”*

#### Accomplishing goals

Many participants discussed feeling successful through accomplishing goals, including longer-term goals like having a family or increased education as well as short-term goals related to their daily roles:

“…*what makes me feel successful would be, daily goals, like making a list and following through. Even, if it is simple as getting some fruit at the store*.”

#### Professional achievement

In addition, their own professional achievement was frequently mentioned as leading to feelings of success. Some participants felt successful because others trust and depend on them, their work was recognized by others, or they are achieving goals such as getting good grades in school. Having sufficient financial resources to meet basic needs and feel secure was also mentioned:

*“One of the things that makes me feel successful is when all of my needs are met and all of my daughter's needs are met. Nobody's going hungry, nobody's walking around naked, and we all got shoes on.”*

While in general, *experiences* from connecting with others and engaging in preferred or self-selected activities contributed to daily feelings of happiness, success seemed to derive from the *outcomes* of their actions. In other words, participants felt successful when they felt competent and effective in their daily roles and responsibilities, especially making a difference in the lives of others. Notably, although participants identified success as coming from accomplishing broader goals, accomplishing mundane daily goals, like getting what they needed at the grocery store, also led to feeling successful.

### Beliefs and feelings about PA

Across the discussions related to PA specifically, five primary themes emerged: (1) Narrow definitions of PA, (2) Feeling pressured, (3) Negative affective recall and forecasting, (4) Positive experiences, and (5) PA is a lower priority than family and work. Beliefs and feelings about PA are where the clearest and most consistent differences emerged between low and high active participants.

#### Narrow definitions of PA

When asked “what counts” as PA, low active participants tended to define PA in ways using a specific and narrow set of standards that traditional PA recommendations have used to educate people about PA, such as signs that they were exerting themselves in high-intensity exercise (e.g., increased heart rate and/or sweating, or feeling “like you’re burning something”) and for the “right” amount of time (e.g., 30 min). Low active participants also described feeling pressured by these criteria and not able to achieve them:

*“You have to do this at this time, and you have to commit to these hours. You have to do this activity. You have to be so good. I feel like it's a lot of pressure for me, with exercise, to perform and do well and commit to that schedule. I can't commit.”*

Women in the low active groups noted that the location, cost, and the hassles of parking at a gym were other barriers to PA, suggesting the belief that PA needs to occur at specific, formal exercise places like gyms. One low active participant said that walking her dog was a barrier to being active, which implied that she did not believe dog-walking counted as valid PA.

High active women were much less likely to make comments that were categorized in this theme.

#### Feeling pressured

In general, low active participants, as well as some participants in one of the two active groups, expressed that they were not exercising as much as they should, want to, or used to. There was a clear sense that they experienced internal pressures related to the idea of being physically active. These participants commonly expressed that they *should* or *need* to engage in PA. Statements reflecting this pressure were often linked to a desire to obtain appearance, weight, or health benefits. For example:

*“I really need to discipline myself and lose the weight again. It's been fluctuating, going up and down, up and down.”*

Losing weight was more frequently discussed as a way to improve their appearance than benefit their health. Both low and high active participants often compared themselves to smaller women or to the size they used to be*,* indicating discrepancies between their current vs. ideal body weight:

*“Before my kids I was size six, size eight. I used to do a lot of sports. Since we moved to this country, life changed a little bit for us….I say that's one of the things I want to work on, on my weight.”*

However, striving to lose weight through PA seemed to be more stressful than motivating, as it was accompanied by self-judgment, ambivalence and negativity toward being physically active:

“*It makes me really resentful when I'm like, "OK, this is you. No one has put weight bags on your body. This is actually your extra weight…when I do exercise now, I have such a bad attitude*.”

#### Negative affective recall and forecasting

Many low active participants acknowledged some degree of *anticipated* negative affect about being physically active. Negative affect was anticipated across all phases of PA: (1) *before* (e.g., just thinking about exercising was stressful), (2) *during* (e.g., feeling self-conscious, cranky, bored, hot, or sweaty), and (3) *following* PA (e.g., feeling soreness, pain, or fatigue). This negativity was apparent through the language used, such as “dreading” exercise, feeling “resentful,” and “*feeling like you’re going to die by the end of [an exercise class].”* In particular, participants in the low active groups talked about experiencing physical discomfort and/or fatigue when they are active, often because of excess weight:

*“I'm not sure because of the age, or because I'm gaining weight* ***.*** *I feel tired just to think about [going] to the gym… that is not fun.”*

Some low active participants discussed that they didn’t want to be physically active because it didn’t deliver the relaxation benefits they desired:

“*When I am already tired, you want to go home and relax, and exercise has nothing to do with relaxation*.”

#### Positive experiences

Although women expressed negative affective associations with PA—particularly those in the low active groups—both low and high active participants also noted positive experiences derived from PA, including factors that motivated and facilitated PA. Being active *with others* was a key ingredient for positive experiences, and was described as creating a sense of community, making it more fun, and providing mutual support and accountability. One high active participant noted that being active with her family is a happiness-generating activity for everyone:

*“Incorporating it in a fun way really helped me out. [My kids] rode their bikes and I ran behind them. That helps big time because it feels like a family. It's good for me. It's good for them. That has actually got me doing it pretty much every day…I feel happier. I think my kids are happier… as much as I am.”*

In addition, contextual factors, such as being active outside on a nice day and/or listening to music, were noted as enhancing the enjoyment of PA. Some participants in both groups discussed PA as leading to more general positive outcomes (e.g., having energy, feeling motivated, and having sense of accomplishment). Others noted specific immediately experienced affective rewards from being active, such as helping to “release stress,” and providing “inner peace” and “a jolt of energy.”

Interestingly, participants in the low and high active groups tended to speak of the positive experiential benefits differently. The low active participants talked about the potential energizing effects of PA as *hypothetical* in nature in that they did not seem sufficiently motivated to seek them out (*“I know I would be healthier and probably happier and less tired if I exercised. Honestly, right now in my life, I’d rather take a nap.”*). In contrast, participants in the high active groups described feelings of success that came from *having already completed* a workout (*“You feel accomplished… You’re breathing a little bit better. It seems like you’re standing a little taller.”*)

#### PA is a lower priority than family and work

Across all focus groups, most reported that their highest priorities were providing for their family, spending time with family, work or school, and meeting financial obligations, the very factors they had previously noted as contributing to their happiness and success:

“*If he [spouse/partner] needs something, if they [children] need something, if my clients need something, all those things come first....Those things are higher priority*.”

As could be expected, the issue of where PA was prioritized differed between low and high active focus groups. Participants in low active groups frequently noted that if they were physically active it would cut into their family time. A few even noted feeling guilty or selfish about taking time away from their families to engage in PA or “me time”:

“*It would be selfish or perceived as selfish if I took the time and [did] not sit down with my family for the dinner time. If I went and did something else and took time for my exercise class or my routine. It's picking your family over time for you*.”

Interestingly, among some low active participants, PA as a means to lose weight or improve health was discussed as a low priority, while for others, it was reported as a top priority. However, those who reported PA for weight loss as a high priority tended to speak about PA using future and conditional language (e.g., “I would”) or what they did in the past rather than what they were currently doing. In fact, one low active participant who was a cancer survivor, noted that although she engaged in regular PA right after her diagnosis, she now (five years later) felt guilty about not taking care of herself or prioritizing PA as much as she used to. Comments about the health benefits from being active suggested a gap between *knowing* about the health benefits from PA and actually *being motivated* by them:

*“…[exercising is] a high priority, just because I know my family history and my genes and all that stuff. It's there telling me that I should do something,* *but it's not a high enough priority for me to actually...*.[trails off].”

Some participants in high active focus groups reported that nothing interfered with their PA because it was integrated into their daily routine. But comments from other high active participants suggested having a more flexible mindset about the role of PA within their daily priorities, noting that some days they will exercise and some they will not, and “*it’s not the end of the world*” if they do not do it on any given day. PA was discussed by these high active participants as a “middle” priority (instead of a top one) permitting compromise and flexibility on any given day.

“*If we have to spend the long nights [helping] my son on a homework assignment, the workout needs to go on the wayside, and so be it….you have give and take*…”

It is also important to note what was not discussed during the conversations about participants’ priorities. Only one participant in all of the groups spoke about where she prioritized *herself*, noting that PA ranks low because she prioritizes *herself* at the bottom.

## Discussion

This is the first study we know of that qualitatively investigated women’s more proximal goals, values, and priorities and the ways in which universal goals such as happiness and success intersect with PA. Many of the concepts that both low and high active participants discussed in this study map on to key principles of SDT, although in different ways for low vs. high active participants when it came to PA. Interestingly, SDT’s posited psychological needs for autonomy, relatedness, and competence [12] appear to underlie feeling happy and successful, as well as being intertwined with and influencing whether physical activity supports or detracts from happiness and success among participants. The discussion is organized around 1) happiness and success; 2) PA in relation to happiness and success; and 3) implications for PA messages and communication strategies. At the end of this discussion, we propose preliminary recommendations about how women’s proximal goals, priorities, and desired experiences could be integrated into new PA messages to better foster women internalizing the value of PA and motivate participation.

### Happiness and success

Participants reported feeling happy when they connected to and were of service to others, were free from daily pressures, felt relaxed and participated in leisure activities they chose to do. This finding aligns with SDT’s contention that autonomy is a primary psychological need and freedom is a core human value [12, 25]. They also described success as being due to effectively performing, meeting goals, and accomplishing daily tasks, suggesting a central need for competence and effectance in their daily lives, as research has suggested [12, 30, 31]. For both happiness and success, participants described connecting with and contributing to the lives of others, reflecting a primary way in which their psychological needs for relatedness are met [32]. This finding aligns with research suggesting that relationships constitute a deeply meaningful and influential aspect of living [32–34], and that feeling related is a fundamental human need and a requisite for well-being [12, 35]. Relationships and connecting with others were the only issues participants reported that fostered *both* happiness and success.

### Physical activity in relation to happiness and success

Interestingly, although both low and high active groups noted similar factors that contribute to feelings of happiness and success, low active and high active participants described notably different beliefs and feelings about PA. High active participants discussed PA as being more congruent with their goals and values, as research suggests is important for goal attainment [36]. The comments from high active participants also suggested that PA supports their psychological needs for autonomy (e.g., flexibility to shift PA to lower priority on days when other demands are high), competence (e.g., experiencing a sense of accomplishment from being physically active), and relatedness (e.g., viewing PA as a way to connect and spend time with important others) [12]. Women in the high active groups were less likely to endorse narrow definitions of PA (i.e., as all-or-none propositions) and have more flexible strategies, which may be indicative of having more internalized motivation toward PA [37] and contribute to the successful self-regulation of health behaviors [18].

**Fig. 1:**
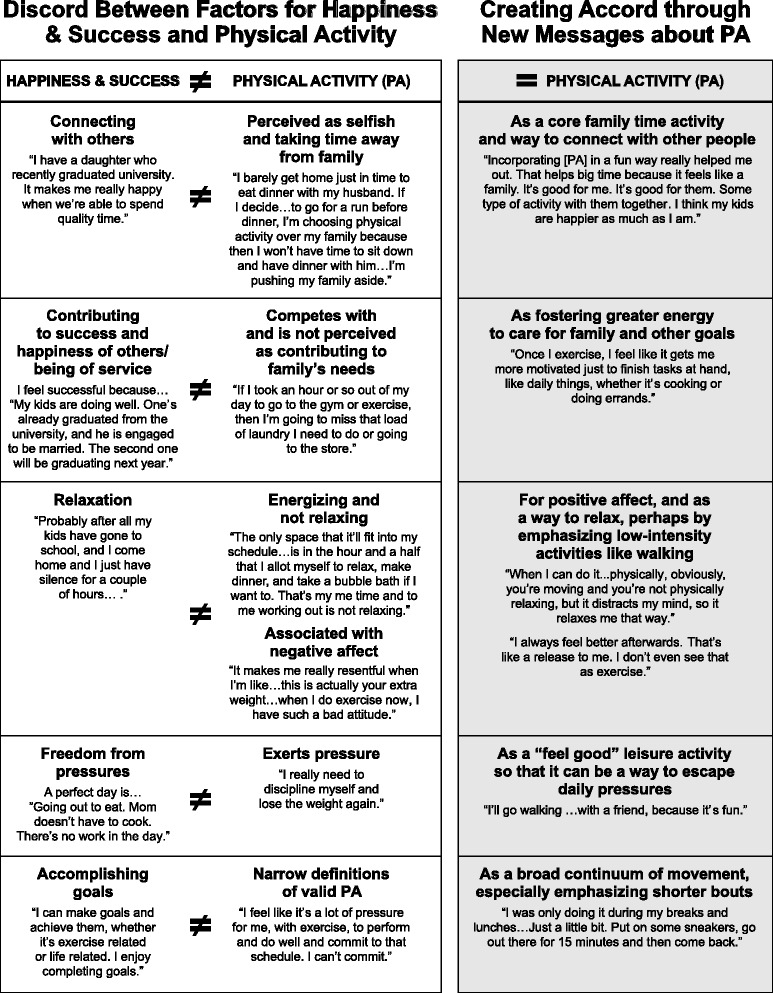
Incongruences between happiness and success and PA, and suggestions for creating congruency through new messages

In contrast to the high active participants, low active participants’ PA beliefs, feelings, and experiences appeared to conflict with and be incongruent with the very goals and experiences that make them feel happy and successful, in addition to thwarting their autonomy, relatedness, and competence. Five specific inconsistencies between happiness/success and PA were identified and are highlighted in Fig. 1, and the primary incongruences are discussed in detail below.

#### Physical activity does not support connection and family

Whereas connecting with and contributing to the success and happiness of others were described as fostering feelings of happiness and success among participants, PA was often described by low active participants as being selfish and taking away from providing for one’s family. This perceived cost of being active could be viewed as thwarting the need for relatedness and likely influences women’s choice to *not* engage in PA [27]. While some participants noted that they felt successful when they were able to meet their family’s basic needs related to clothing, food, and shelter, these types of basic survival needs are often not considered in PA messaging. When women are concerned about meeting their family’s needs (basic and otherwise), PA messages that do not explicitly address these core concerns may make PA irrelevant to them.

#### Physical activity undermines how women want to feel

Although many participants described happiness from experiencing states of relaxation and recovery, they often described PA as the opposite: requiring high levels of exertion, thus thwarting their sense of autonomy toward being physically active. Participants also tended to describe PA as leading to energization rather than the relaxed states they desired. Many low active participants also anticipated negative affect from being active, such as dread, fatigue, or pain. This negativity is likely very influential in decisions not to participate in PA given that anticipated affect has strong motivational properties [38, 39] and shapes behavioral choice [40].

Furthermore, whereas participants desired time to be *free from pressures*, especially of daily responsibilities and schedules, low active participants often discussed *feeling pressured by* the need to meet the standards of exertion and duration needed for what they *perceived* to be valid PA. PA was perceived by many low active participants as a chore, something they *should* do, and as a source of guilt that they did not do it, reflecting a controlled, non-optimal form of regulation. Pressured feelings about PA may be further exacerbated by low active participants’ general view that they should be physically active in order to lose weight and improve their health (e.g., introjects), often negatively comparing themselves to other women and themselves when they were thinner [37, 41]. Given that women have limited time to participate in leisure activities [42], PA that is experienced as pressurizing, associated with poor body image, or generates negative affect is an unlikely candidate for low active women’s cherished “off time” [43]. Nor is it likely to motivate the consistent self-regulation needed for sustainable PA [18].

#### Physical activity criteria make it too hard to successfully fit in

Participants stated that accomplishing goals led to feelings of success. Yet the narrow ways in which low active participants defined PA not only pressured them, they undermined the likelihood of successful feelings from being physically active because they reported not having sufficient time or energy to achieve those specific criteria [42], likely thwarting their need for competence. These data suggest that the last 20 years of public health messaging about PA may have failed to effectively convey the value of activities of daily living (e.g., dog-walking, taking the stairs) and more moderate-intensity PA. Indeed, a large nationally representative survey showed that less than 1% of American adults understand the current, more moderate-level PA recommendations [44].

### Implications for PA messages and communication strategies

These findings offer new insights into specific ways women’s more proximal goals and priorities can be integrated into physical activity messaging to better foster the internalization of the value of PA and motivate participation among low-active ethnically diverse women. Supporting other research among women [42], these findings suggest a need to address the expectations and pressures that women experience which undermine PA participation. In addition, new messages should help women understand that PA can facilitate rather than compete with their daily roles, priorities, and desired positive affective experiences [45]. These findings further suggest the need to expand both the role and definition of valid PA so women can feel more able and competent fitting it into their complex lives. The last column in Fig. 1 highlights specific ways in which PA messages could become more congruent with women’s daily goals and values, particularly the experiences from which they derive a sense of happiness and success. Below are four overarching preliminary suggestions to consider in future PA communication research and evaluation.

#### Promote PA as a way to connect with others

Connecting with others was the only factor participants described as facilitating *both* success and happiness, suggesting it has potential to be a compelling benefit from and motivator for PA. This finding is consistent with research showing that being connected to others is associated with happiness and meaning [33, 34], and with SDT’s identification of relatedness as a key psychological need [12]. Furthermore, being active *with* others was specifically noted by participants as making PA a positive experience. Thus, not only might being active with others satisfy needs for relatedness, research suggests relational motivation is important for PA [46], especially among women [47]. Interestingly, only one person in all of the focus groups mentioned feeling happy from connecting with others online. Given the recent emphasis on using online communities and social networking to motivate physical activity [48, 49], it is important to study whether in-person compared to online connections have similar or different relational benefits and how best to leverage these different types of connections to foster PA motivation and participation.

#### Reframe PA as congruent with women’s roles and responsibilities

Low active participants especially reported PA as a low priority and often felt guilty and selfish about taking time away from their family duties to be physically active. Multiple roles and responsibilities combined with an ethic of care creates role strain, and is a significant barrier to PA for women [42]. Therefore, messaging that emphasizes that time spent in PA can contribute to, rather than detract from, family and work responsibilities [50] may help PA be viewed as facilitating women’s top daily priorities and goals (i.e., goal facilitation) [51]. In addition, messages that frame engaging in acts of self-care, like PA, as a way to better equip women to deal with the various other demands in their lives (e.g., having more energy for work, resiliency in the face of challenges) may help low active women more easily give themselves permission to take time to be physically active [50, 52]. Having messages align PA with women’s most salient goals and priorities should foster self-congruence and thus support women better internalizing the value of being physically active; resulting in more internalized forms of motivation and greater self-regulatory persistence in the face of barriers [18, 36, 53].

#### Emphasize PA as a way to generate positive affect

Participants reported wanting experiences that are relaxing and time that is free from pressure. Given this, they might be more likely to view PA as contributing to, rather than in conflict with, these desired experiences if they believed that PA will generate positive and restorative experiences [54]. Thus, new PA messages could provide clearer guidance on self-determined exercise intensity for low active women, recommending that they start at intensities that generate immediate positive affect *for them.* This strategy should make PA more pleasurable [55], intrinsically motivating [56], and also support women’s needs for autonomy and choice over PA [15], all of which, should increase the likelihood that they will internalize the value of being physically active and become more physically active [12, 57–59]. Furthermore, because inactive individuals also tend to anticipate less positive affect from PA than active individuals [60], increasing expectations of positive feelings from exercise is likely to foster greater post-exercise positive affect and intentions to exercise [54].

In addition, participants in both groups felt generally pressured about being active, especially in relation to their current size and desire to lose weight, and to improve their health. Given that motives related to appearance and weight are associated with greater self-objectification, controlled regulation, negative affect, and less participation than non-weight/appearance related motives [61–63], it might be especially important for new messages targeting women to reframe the purpose of PA away from weight and appearance to positive affect and daily well-being [64]. Similar to other research [65], it is important to note that while participants knew about the health benefits from PA, this knowledge did not actually motivate them to be physically active.

#### More effectively communicate a broad continuum of physical activity

Low active participants defined PA using a narrow set of criteria that emphasized the need to be active at high intensities and for longer durations that reflected older PA recommendations (e.g., 45 min). Yet, similar to what has been found in previous research [42], they reported being unable to achieve these criteria due to time constraints and low energy levels. Furthermore, most did not discuss or endorse more moderate-level activities of daily living as *valid* PA options. These findings suggest a need for public health communicators to better convey the value of lifestyle, lower-intensity, and shorter-duration bouts of activity, especially to women [42]. Thus, messages emphasizing the accumulation of lifestyle bouts of movement might make PA feel less like a competing priority because shorter bouts can be more seamlessly integrated within the natural spaces that exist in their daily lives.

Lack of competence/self-efficacy toward PA is a known barrier to participation among women [66]. Therefore, promoting a broader continuum of PA encourages women to move whenever they can and should foster competence and feelings of success whenever they participate in *any* physical movement. In addition, some participants identified success as coming from accomplishing mundane daily goals, such as getting what they needed at the grocery store. Similarly, helping women believe in the value of doing small bouts of activity should foster feelings of success and be self-reinforcing. This new messaging strategy suggestion also supports autonomy because it gives women permission to engage in PA in flexible ways [18] that works for them *at any given moment*.

This new message advocating women move more during the day in shorter, more convenient bouts is supported by research showing that sitting leads to the precursors of diseases like diabetes [67] and that the physiological health benefits of movement quickly decline [68], underscoring the importance of moving consistently and frequently throughout the day. If PA promoters can help low active women learn that “everything counts,” and that any movement is better than no movement, they might start to seek out easy-to-integrate bouts of movement in their daily lives. This might also help PA become a more automatic behavior.

#### Limitations and strengths

Limitations of the study include the small sample size. However, because there were three times as many low active as high active participants, the findings about differences between these groups are tentative and in need of additional support. Despite this, the discussions suggested that differences existed between low and high active groups regarding their PA beliefs, feelings, and definitions. In addition, our study participants were more highly educated than the general population. Future studies can use ethnographic observations to help identify potential spaces and windows of opportunities to move that exist in women’s days.

This study also has strengths. These data come from an ethnically diverse sample of women. Further, this is the first study we know of that has conducted qualitative research about physical activity within the context of women’s daily goals, values, and priorities to identify the ways in which PA supports and/or thwarts these goals and values. Thus, this is an important formative step toward understanding how to develop more compelling and persuasive PA communication strategies for women.

## Conclusions

People’s beliefs, goals, and priorities derive out of culture norms and are an expression of the self; they strongly influence people’s daily behavioral choices [12, 30]. Striving towards goals that are personally meaningful and self-congruent is associated with high-quality motivation, persistence in the face of challenges, and well-being. This study provides interesting new insights about women’s salient goals, priorities, and desired leisure-time experiences and the ways in which they support and are in conflict with being physically active. Interestingly, although both low and high active participants noted similar goals and experiences related to happiness and success, they described notably different beliefs and feelings about PA. In general, low active participants’ beliefs and feelings about and definitions of PA reflected the opposite of and were incongruent with the factors that delivered happiness and success. PA messages influence women’s perceptions about what PA means and the role it plays in their lives [5]. Therefore, rather than emphasize the goals public health professionals value, messages should align PA with the experiences, goals, and priorities that *women care most about* [69] in order to get their attention in the crowded communications environment [11] and make PA more relevant, pleasurable, and easier to integrate into their daily lives [70, 71]. These insights suggest fertile ground to investigate more strategic and persuasive approaches to communicate about PA in ways that can better motivate the prioritization of PA participation and improve public health.

## Abbreviations

IRB: Institutional Review Board
PA: Physical activity
SDT: Self-Determination Theory
